# The undertranslated transcriptome reveals widespread translational silencing by alternative 5' transcript leaders

**DOI:** 10.1186/gb-2005-6-13-r111

**Published:** 2006-01-03

**Authors:** G Lynn Law, Kellie S Bickel, Vivian L MacKay, David R Morris

**Affiliations:** 1 Department of Biochemistry, University of Washington, Seattle, WA 98195, USA

## Abstract

**Background:**

Translational efficiencies in *Saccharomyces cerevisiae *vary from transcript to transcript by approximately two orders of magnitude. Many of the poorly translated transcripts were found to respond to the appropriate external stimulus by recruiting ribosomes. Unexpectedly, a high frequency of these transcripts showed the appearance of altered 5' leaders that coincide with increased ribosome loading.

**Results:**

Of the detectable transcripts in *S. cerevisiae*, 8% were found to be underloaded with ribosomes. Gene ontology categories of responses to stress or external stimuli were overrepresented in this population of transcripts. Seventeen poorly loaded transcripts involved in responses to pheromone, nitrogen starvation, and osmotic stress were selected for detailed study and were found to respond to the appropriate environmental signal with increased ribosome loading. Twelve of these regulated transcripts exhibited structural changes in their 5' transcript leaders in response to the environmental signal. In many of these the coding region remained intact, whereas regulated shortening of the 5' end truncated the open reading frame in others. Colinearity between the gene and transcript sequences eliminated regulated splicing as a mechanism for these alterations in structure.

**Conclusion:**

Frequent occurrence of coordinated changes in transcript structure and translation efficiency, in at least three different gene regulatory networks, suggests a widespread phenomenon. It is likely that many of these altered 5' leaders arose from changes in promoter usage. We speculate that production of translationally silenced transcripts may be one mechanism for allowing low-level transcription activity necessary for maintaining an open chromatin structure while not allowing inappropriate protein production.

## Abstract 2

Eight per cent of yeast transcripts, mostly involved in responses to stress or external stimuli, were found to be under-loaded with ribosomes, and most of them exhibited structural changes in their 5’ transcript leaders in response to the environmental signal.

## Background

Across a cellular transcriptome the loading of ribosomes onto individual mRNA species varies broadly [1-3], consistent with each transcript having a uniquely defined efficiency of translation. Translational efficiencies across the transcriptome of *Saccharomyces cerevisiae *have been estimated to vary from transcript to transcript by approximately two orders of magnitude (as reported by MacKay and coworkers [3] and herein). Many factors contribute to transcript-specific translation efficiencies, including those intrinsic and extrinsic to mRNA structure [4]. Extrinsic factors include regulation of the activities of translation initiation factors through phosphorylation [5,6] and regulation of the binding of transacting molecules [7-9]. Factors intrinsic to the specific mRNA include features of the 5' untranslated region (UTR) that inhibit ribosome scanning such as secondary structure [10] and upstream open reading frames (ORFs) [11]. In addition, altered translational efficiency can arise from regulated changes in mRNA structure, such as modifications in transcript structures occurring through alternative use of promoters and splice sites within the nucleus [12], as well as RNA splicing and polyadenylation mechanisms occurring in the cytosol [13,14]. The relative importance of these various regulatory mechanisms differs widely from transcript to transcript in a given cell or tissue.

In the present study, we identified a set of under-translated transcripts of *S. cerevisiae*. Within this group of transcripts, we found over-representation of the Gene Ontology (GO) categories related to environmental responses of the organism, suggesting that mRNA translatability may be controlled in response to exogenous stresses. Transcripts from three of these GO categories, namely responses to pheromone, nitrogen starvation, and osmotic stress, were selected to test this hypothesis. Many of the under-translated transcripts selected were found to respond to the appropriate environmental signal with a change in ribosome loading. Remarkably, we found that a majority of these alterations in translation are accompanied by a change in the 5' UTR of the transcript. These findings suggest that changes in translational efficiency as a consequence of altered transcript structure are much more common than was previously suspected. Furthermore, those alterations that arise from changes in promoter usage have implications with regard to the fate of intergenic transcripts involved in regulation of gene expression.

## Results

### The under-translated transcriptome

**Figure 1:**
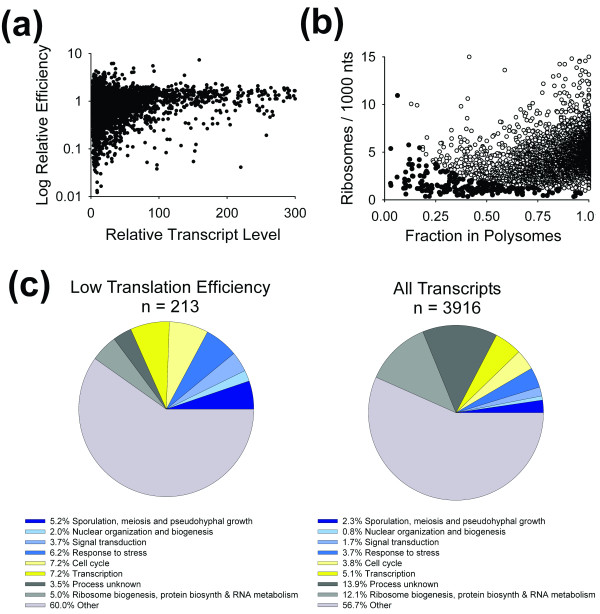
The under-translated transcripts of *Saccharomyces cerevisiae*. **(a) **Translational efficiency across the transcriptome. Translation state array data from exponentially growing yeast were used for 3916 transcripts with open reading frames (ORFs) longer than 400 nucleotides and whose distributions after sucrose gradient centrifugation could be modeled reliably [3]. To calculate translational efficiency, the fraction of each transcript in polysomes was multiplied by the mean ribosome density, expressed as ribosomes per 1,000 nucleotides of ORF, and these values were normalized to a mean of 1.0. Translational efficiencies are plotted on a logarithmic scale versus relative transcript level obtained from the array analysis [3]. **(b) **Ribosome loading on the transcriptome of steady-state growing yeast. Ribosome density (ribosomes per 1,000 nucleotides) is plotted against the fraction of each transcript in polysomes. The data are those used to calculate the translational efficiencies in (a). The under-translated transcripts (<0.25 of the mean translation efficiency) are represented by the filled symbols and the remaining transcripts by the open symbols. **(c) **Gene ontology (GO) analysis of the under-translated transcriptome. The frequency of biologic process catagories appearing in the indicated populations was analyzed, as described in the text, using the GO tools associated with the *Saccharomyces *Genome Database [66].

Sucrose-gradient centrifugation, coupled with genome-wide transcript measurements, has enabled genome-level analysis of ribosome loading on individual transcript species [1,3]. Measurements of the fraction of a given transcript associated with polyribosomes, together with the average spacing of ribosomes along the mRNA, allows estimation of the efficiency of translation and hence the rate of synthesis of the encoded protein [3]. Translational efficiencies calculated across the transcriptome of growing yeast are presented in Figure 1a. The diversity of association of individual transcripts with the translational apparatus is apparent from these values for translational efficiencies. These quantities vary by more than two orders of magnitude, illustrating dramatically the unique translational properties of each individual transcript species.

For the purposes of subsequent analysis, those transcripts with translation efficiencies below 0.25 of the mean were arbitrarily defined as under-translated. By this definition, of the 3,916 transcripts for which reliable polysome profiles could be modeled, fewer than 10% (298 transcripts) were found to be under-translated [3]. Two experimentally accessible characteristics combine to achieve inefficient translation of these transcripts: the fraction of a transcript in the act of being translated (for example, associated with ribosomes) and the average spacing of ribosomes along a translating mRNA. Across the entire transcriptome, the average fraction of transcripts associated with ribosomes is 0.82 and the average ribosome density is 4.4 ribosomes per 1,000 nucleotides. For most members of the under-translated transcriptome, both parameters lie below these population means (Figure 1b, filled symbols). At the extremes of the distribution, a few of the under-translated transcripts are more than 90% associated with ribosomes but sparsely loaded. Likewise, a few others possess ribosome densities that are average or above, but with less than 20% of the transcripts actually present in polysomes.

In the under-translated transcriptome, 213 of the 298 transcripts are the products of named genes. The biologic processes associated with this poorly translated group are explored in Figure 1c. Because the analysis was restricted to just the subset of named genes, the category 'process unknown' represents only 3.5% of this selected group of transcripts, in contrast to 13.9% in the complete dataset. The GO categories significantly (*P *< 0.01) over-represented or under-represented in the under-translated transcriptome are specifically broken down in the figure, whereas all others are combined in the 'other' category. The processes of protein synthesis, ribosome biogenesis, and RNA metabolism are under-represented in the under-translated transcriptome, which was expected because the transcripts analyzed were derived from steady-state growing cells, where protein synthesis is vigorous. In contrast, responses to environmental changes such as 'response to stress', 'cell cycle', 'signal transduction', and 'sporulation, meiosis and pseudohyphal growth' were significantly over-represented in the population of under-translated transcripts. Individual representatives from these environmental response categories were selected from the under-translated population, and their responses to external stimuli were evaluated.

### Translational responses of the transcriptome to mating pheromone

**Figure 2:**
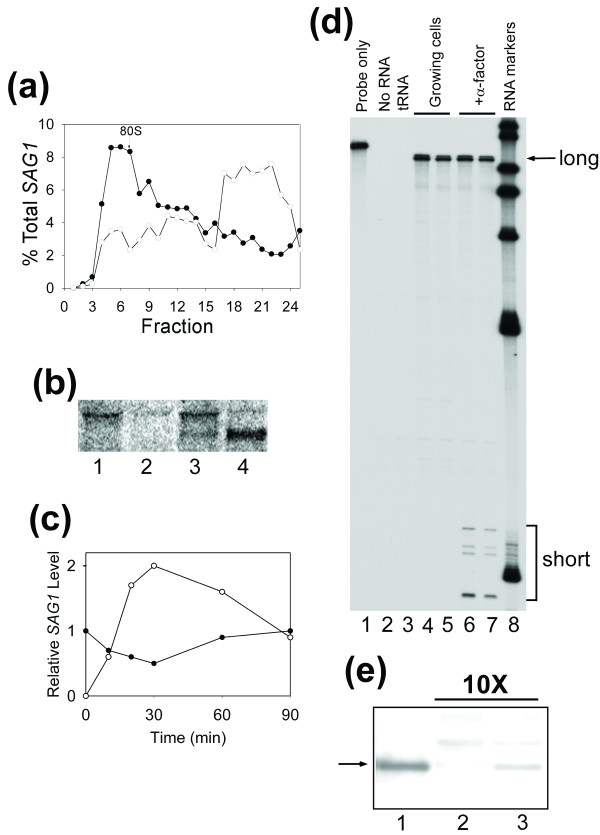
Structure of the 5' leader of the *SAG1 *transcript regulates its translation. **(a) **Distribution of *SAG1 *mRNA across polysome gradients in growing cells (filled circles) or cells treated with a-factor for 30 min (open circles). Cell lysates [3] were loaded onto 7-47% sucrose gradients and spun for 1.5 hours in a SW40 rotor at 39,000 rpm at 4°C. Levels of *SAG1 *transcript in each gradient fraction were determined by real-time polymerase chain reaction (QPCR) and the signal in each fraction was divided by the sum of the signals in all fractions. The top of the gradient is to the left and the position of the 80S monosome is marked with the arrow. **(b) **Northern blot analysis of *SAG1 *RNA from growing cells (lanes 1 and 2) or from cells after 45 min of a-factor treatment (lanes 3 and 4). Equal cell equivalents of RNA from pooled sucrose gradient fractions 1-14 (lanes 1 and 3) or pooled fractions 15-25 (lanes 2 and 4) were analyzed. **(c) **Relative levels of *SAG1 *mRNA at different time points after treatment with a-factor. Total RNA was isolated from cells treated with a-factor for the indicated times and cDNA was produced with reverse transcription by priming with oligo(dT)_25_. *SAG1 *transcript levels were determined by QPCRusing either primers recognizing both transcripts or primers specific for the long transcript. Closed circles show the values for the long form and the open circles represent calculated values for the short *SAG1 *transcripts, computed as the differences between the values for both transcripts and those for the long transcript. The curves are normalized to a value of 1.0 for the long transcript at zero time of treatment. **(d) **RNase protection assay showing two forms of *SAG1 *: lane 1, probe only; lane 2, no RNA; lane 3, tRNA control; lanes 4 and 5, 50 µg total RNA from growing cells; lanes 6 and 7, 50 µg total RNA from cells treated for 30 min with a-factor; and lane 8, RNA markers. The antisense RNA probe was prepared from cloned genomic sequence and contained 55 nucleotides of open reading frame, 484 nucleotides of 5' leader, and 92 nucleotides of noncomplementary sequence. Independent RNA preparations were used in lanes 4-7. Locations of the protected probes corresponding to *SAG1 *long 5' leader (539 nucleotides) and short 5' leader (95 nucleotides) are indicated. **(e) **Western blot analysis of protein extracts from growing cells was performed to determine the relative levels of His3-HA protein from yeast strains transformed with reporter constructs containing the *ADH1 *promoter and *SAG1 *short 5' leader (lane 1), *SAG1 *long 5' leader (lane 3), or the empty vector (lane 2). The arrow indicates location of the His3-HA protein. As indicated in the figure, lanes 2 and 3 had 10 times more protein loaded than did lane 1.

Previously, in a genome-level analysis of the response of yeast to a-factor, we found 163 transcripts that increased in ribosome loading and 36 that decreased [3]. From this previous study, we selected eight regulated transcripts for detailed examination, along with three control genes, which increase in transcript level in response to pheromone but maintain constant ribosome loading (Table 1). *SAG1 *encodes a surface protein that is important for cell-cell interaction during mating [15,16] and, in growing cells, a large fraction of the population of *SAG1 *transcripts is poorly loaded with ribosomes, as assessed by sucrose gradient centrifugation (Figure 2a). After pheromone treatment, ribosome loading on this mRNA was enhanced, coincident with the appearance of a new, short form of the *SAG1 *transcript in Northern blots, which was undetectable before treatment and is strongly localized in polysomes (Figure 2b, lane 4). This efficient loading of the short transcript with ribosomes was in clear contrast to the long form, which is found to sediment primarily with mRNP particles and monosomes in the presence or absence of pheromone (Figure 2b, lanes 1 and 3). The poor loading of the long transcript was confirmed by real-time polymerase chain reaction (QPCR) using primers specific for this form (data not shown). The short, well translated *SAG1 *mRNA reached a maximum level double that of the long transcript at 20-30 min after exposure to a-factor (Figure 2c).

RNase protection assays (Figure 2d) revealed that the long *SAG1 *transcript has a 5' end greater than 484 nucleotides upstream of the ORF. The short form exhibits a ladder of protected fragments (Figure 2d), probably resulting from either multiple, closely placed transcriptional starts or breathing of the RNA double helix during the assay. The size of the predominant short species is consistent with the 5' end being located at approximately -40 nucleotides relative to the start of the ORF. Results of 5' rapid amplification of cDNA ends (RACE; Table 1), performed on total RNA from either growing cells or cells treated with a-factor for 30 minutes, revealed major 5' termini at positions -826 and -38. Therefore, RNase protection and 5' RACE are consistent with both transcripts containing the initiation codon for the known form of Sag1 protein. The size of the short transcript is consistent with the presence of a pheromone-response element [17] and a TATA box [16] in this region of the genome.

Exploring further the apparent difference in translational efficiency between the two *SAG1 *transcripts, His3p tagged with the HA epitope was used as a reporter [3] in constructs containing either the 826-nucleotide or 38-nucleotide 5' leader of *SAG1 *under the control of a heterologous constitutive promoter. Western blot analysis revealed much higher levels of protein produced from the construct with the short 5' leader (Figure 2e; compare lanes 1 and 3). Because the same protein was produced from both transcripts, the difference in level must have resulted from altered rates of synthesis rather than differences in protein stability. Transcript levels were determined using QPCR (data not shown) and the calculated translation efficiency (protein/mRNA) of the transcript with the short *SAG1 *leader was found to be 4.9 times that of the long *SAG1 *construct, which is consistent with the qualitative assessment of ribosome loading by sucrose gradient centrifugation (Figure 2a, and Table 1). Thus, production of a new transcript with elevated translational efficiency amplifies the protein response resulting from transcriptional induction of the *SAG1 *gene (Figure 2c).

**Figure 3:**
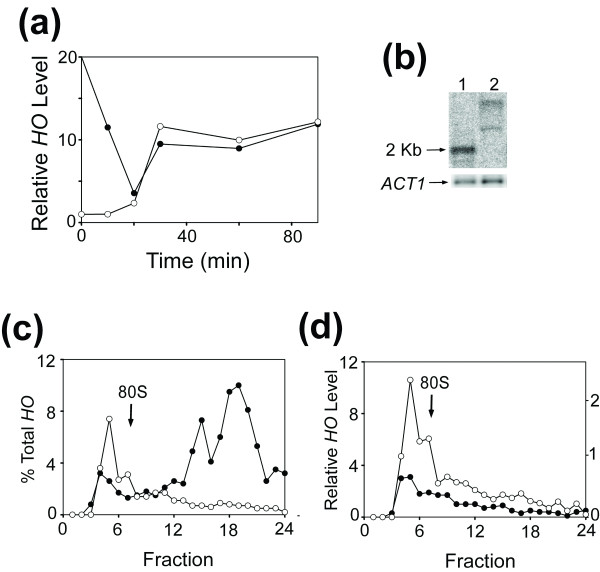
Transcriptional and translational downregulation of *HO *expression in response to mating pheromone. **(a) **Relative levels of *HO *mRNA (normalized to 1.0 for the long transcript at time = 0 min) as a function of time after a-factor treatment. RNA was prepared and analyzed as in Figure 2c. Closed circles show values for both forms of transcripts and open circles represent values for the long *HO *transcript. **(b) **Northern analysis of total RNA (10 µg) from growing cells (lane 1) or cells treated with a-factor for 30 minute (lane 2). The blot was stripped and re-probed for *ACT1 *as a loading control. **(c) **Relative levels of *HO *mRNA across polysome gradients in growing cells (filled squares) or cells treated with a-factor for 30 minute (open circles). Gradients were performed and analyzed as described in Figure 2 using PCR primers recognizing both *HO *transcripts. The top of the gradient is to the left and the position of the 80S monosome is marked with the arrow. **(d) **Relative levels of the long forms of *HO *across polysome gradients in growing cells (filled circles, right axis) or cells treated with a-factor (open circles, left axis). QPCR using primers specific to the long forms of *HO *was performed on cDNA obtained from the same RNA samples used in the experiment described in panel c of this figure. Note the difference in scale on the two axes.

The *HO *gene encodes an endonuclease that mediates switching of mating type in *S. cerevisiae *[18-20]. As previously shown [21], the level of the cell-cycle regulated *HO *transcript precipitously decreased after exposure to mating pheromone; under the experimental conditions employed here, the transcript reached its nadir by about 20 minutes after initial exposure (Figure 3a). Northern blots revealed the expected 2-kilobase form of the *HO *transcript in growing, untreated cells. However, this form was replaced after pheromone treatment by multiple transcripts over 2.5 kilobases in length (Figure 3b). RNase protection assays established that the long forms of the *HO *transcript have 5' leaders that are contiguous with the genomic sequence and extended by more than 470 nucleotides beyond the 5' end of the short transcript (not shown). This change in structure of the transcript produced was accompanied by a profound reduction in ribosome loading on the *HO *transcripts present after pheromone treatment (Figure 3c). QPCR across sucrose gradients, using a primer set specific to the long forms, demonstrated that the long, pheromone-induced transcripts are extremely under-loaded with ribosomes (Figure 3d). Very low, but significant, levels of the long forms are detected in untreated cells and are likewise translated inefficiently. Thus, like *SAG1*, a new *HO *transcript appears upon pheromone treatment. In contrast to *SAG1*, the new form is poorly loaded with ribosomes, which together with decreased transcript level, ceases production of the endonuclease in preparation for mating.

Other transcripts in addition to *HO *and *SAG1 *were found to change their association with ribosomes in response to mating pheromone [3]. These include *CRH1*, *KAR5*, *PRM2*, *PRP39*, and *PRY3*, which - like *HO *and *SAG1 *- all show concomitant alterations in their 5' leaders (Table 1). Interestingly, the poorly loaded forms of these particular transcripts all have their 5' termini located within the protein encoding regions, precluding synthesis of the full-length proteins (see Discussion, below).

**Figure 4:**
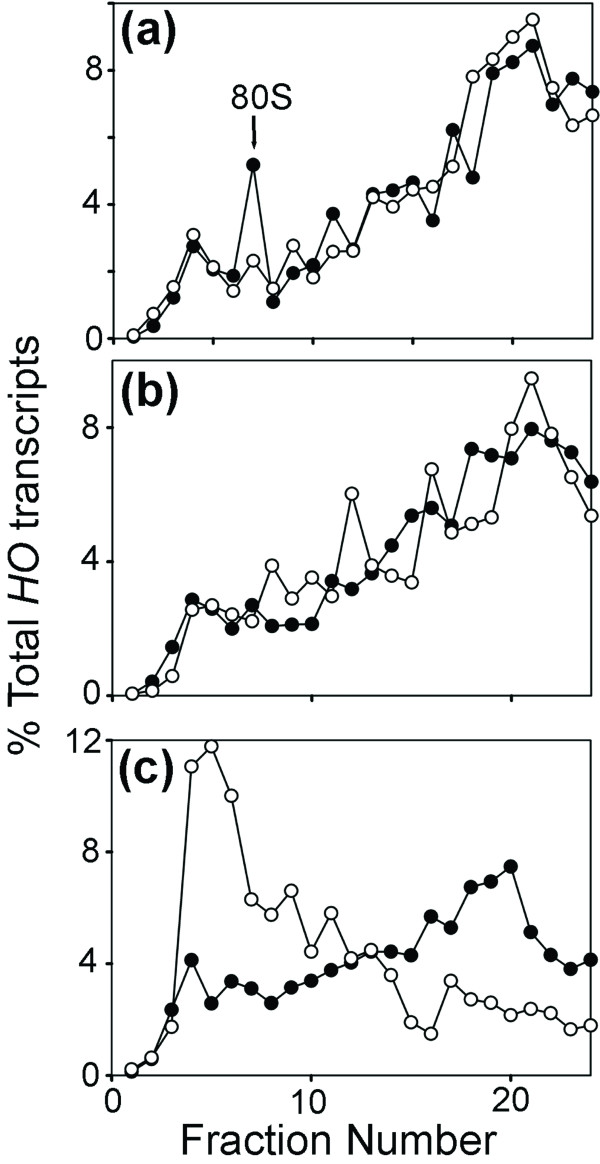
Influence of mutations in the pheromone signaling pathway on translational responses of the *HO *transcript. The top of the gradient is to the left and the position of the 80S monosome is marked with the arrow. Percentage of total *HO *mRNA across polysome gradients in growing cells (filled circles) and cells treated with a-factor for 30 minutes (open circles) for strains **(a) **Δfus3 Δkss1, **(b) **Δste12, and **(c) **parental BY2125. Sucrose gradient centrifugation was performed and analyzed as described in Figure 2, using polymerase chain reaction primers that are common to all *HO *transcripts.

The signal transduction pathway for the pheromone response is well understood, and strains with deletions of the involved genes are viable but do not mate [22,23]. Key components of this pathway are the partially redundant protein kinases Fus3p and Kss1p along with the transcription factor Ste12p. Strains lacking either the two kinases (Figure 4a) or the transcription factor (Figure 4b) exhibited none of the changes in ribosome loading on the *HO *transcript that were seen with the wild-type strain in response to a-factor (Figure 4c; also see Figure 3c). This lack of response of the double *fus3 kss1 *and the *ste12 *deletion strains was also observed with the *SAG1*, *CRH1*, and *PRY3 *transcripts (data not shown). Therefore, it seems that the alterations in ribosome loading on these five transcripts require the entire pheromone signal transduction pathway, including activation of the Ste12 transcription factor. Northern blot analysis of *SAG1*, *CRH1*, and *PRY3 *revealed no change in transcript structure in the *ste12 *mutant, which is consistent with the relationship between 5' UTR structure and ribosome loading.

Many genes respond to a-factor with increases in transcript level, but corresponding alterations in transcript structure were not universally found. For example, four genes - *BAR1*, *FAR1*, *PRM4*, and *STE2 *- all exhibited elevated transcript levels after exposure to a-factor, but none of these showed a modified 5' leader (Table 1). Of these four genes, only *PRM4 *exhibited significantly altered ribosome loading [3], and this transcript is seemingly 'poised' to respond rapidly at the translational level to pheromone. It should be emphasized that, of the pheromone-responsive cohort of genes examined in this paper, *PRM4 *is the only one that showed a change in ribosome loading with no concomitant change in transcript structure.

### Influence of nitrogen starvation on the translation state of the transcriptome

In the collection of under-translated transcripts, 20 were related to responses of yeast to nitrogen starvation (Table 2). Many of these genes encode regulators of nitrogen metabolism and enzymes that are involved in metabolism of secondary nitrogen sources. A subclass abundantly represented in this group contains genes that are involved in the vacuolar process known as autophagy. Through regulated proteolysis of cytosolic proteins [24,25], autophagy liberates the amino acids necessary for synthesis of new proteins required for adaptation to a new nutritional environment.

**Figure 5:**
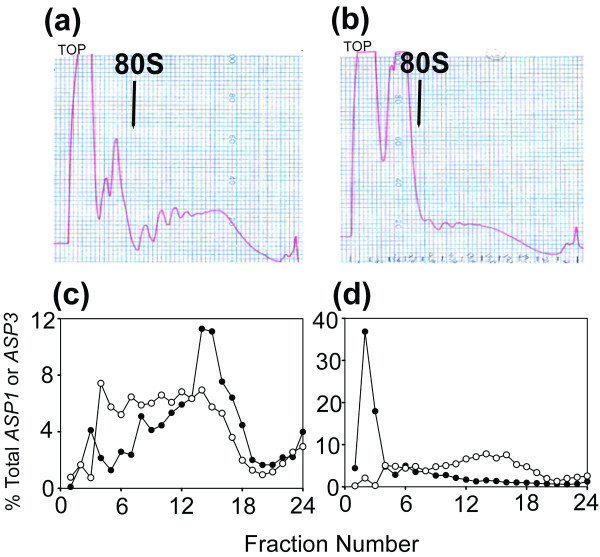
Translational responses to nitrogen starvation. Sucrose gradient centrifugation was performed and analyzed as described in Figure 2. The A_254 _profiles are shown of sucrose gradients with extracts from either **(a) **growing cells or **(b) **starved cells loaded onto gradients. The tops of the gradients and location of the 80S ribosome peak in panel a are indicated. **(c) ***ASP1 *mRNA levels across sucrose gradients from growing cells (filled circles) or cells nitrogen starved for 30 minutes (open squares). RNA was prepared and analyzed as described in Figure 2. The top of the gradient is to the left and the position of the 80S monosome is marked with the arrow. **(d) ***ASP3 *mRNA levels; cell extracts and symbols are as in (c).

Nitrogen starvation causes a generalized inhibition of protein synthesis initiation, probably through activation of protein kinase Gcn2p, which phosphorylates the a-subunit of the key translation initiation factor eIF-2 [5]. In response to transfer of cells to nitrogen starvation medium, there is a programmed loss of polysomes and a concomitant accumulation of free ribosomes (Figure 5; panels a and b). Coincident with the loss of polysomes during nitrogen stress is a general movement of transcripts to smaller polysomes. This is illustrated in Figure 5c for *ASP1*, which encodes a constitutive cytosolic asparaginase. Three other control transcripts that were examined - *GDH1*, *DED1*, and *ERG11 *- all showed the same reduction in ribosome loading as did *ASP1 *(not shown). In contrast to *ASP1*, transcripts from the four identical copies of *ASP3*, which encode the periplasmic asparaginase responsible for utilizing asparagine as a general nitrogen source, become better loaded with ribosomes in response to nitrogen starvation (Figure 5d).

**Figure 6:**
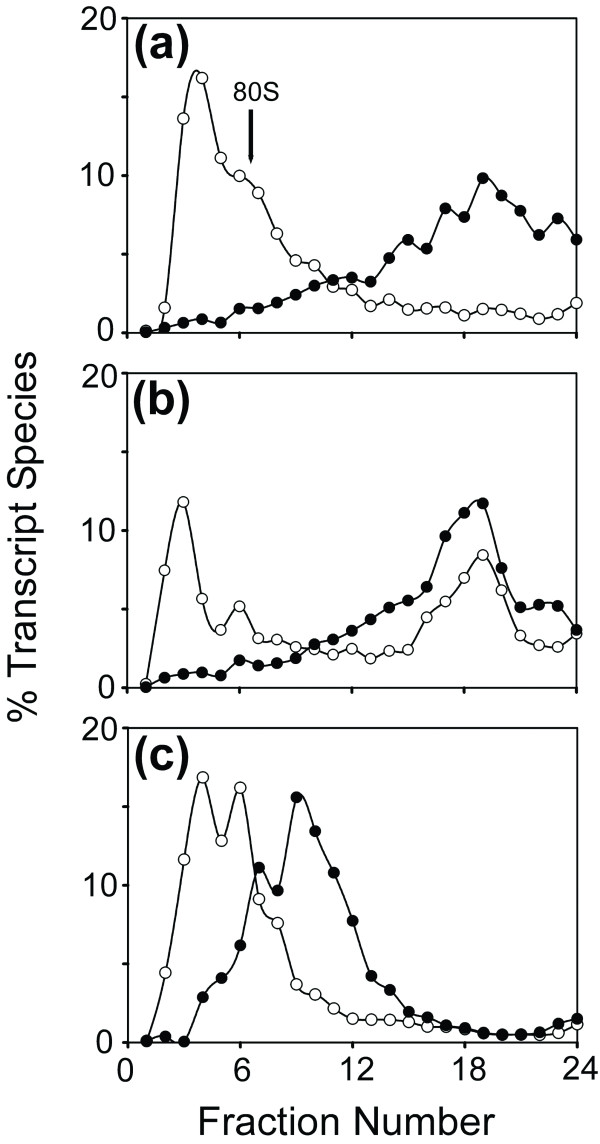
Changes in ribosome loading in response to nitrogen starvation. mRNA levels across sucrose gradient from growing cells (open circles) and from cells nitrogen starved for 30 minutes (filled circles) for **(a) ***DAL5*, **(b) ***UGA1 *and **(c) ***GCN4*. RNA was prepared and analyzed as described in Figure 2. The top of the gradient is to the left and the position of the 80S monosome is marked with the arrow. The experiments shown in (a) and (b)were performed with strain LL1 (Δ*gcn2 *; described in Materials and methods) and the experiment in panel c was conducted with the wild-type strain.

Three other examples of transcripts that run counter to general protein synthesis and become better loaded with ribosomes during nitrogen stress are shown in Figure 6. The *DAL5 *gene encodes an enzyme that is involved in the utilization of allantoin, a secondary nitrogen source for yeast; *UGA1 *encodes a transaminase involved in the catabolism of ?-aminobutyric acid; and *GCN4 *encodes the bZIP protein Gcn4p, which mediates general transcriptional control over amino acid biosynthesis in yeast. These three transcripts exhibit a pattern similar to that seen with *ASP3 *(Figure 6). The activation of *GCN4 *translation in response to amino acid starvation is mediated through the phosphorylation of eIF-2 [5]. Interpretation of the *GCN4 *finding (Figure 6c) is either that the level of uncharged tRNA elevates sufficiently to activate the Gcn2p protein kinase under these conditions of general nitrogen stress [5] or that the state of phosphorylation of Gcn2p itself is lowered as a result of nitrogen starvation [26]. Activation of ribosome loading on the *DAL5 *and *UGA1 *transcripts does not depend on Gcn2p, because the experiments illustrated in Figure 6 panels a and b were performed with a *gcn2 *deletion strain.

The 5' termini of eight transcripts related to nitrogen stress were examined before and after starvation (Table 1). The *ASP1 *and *GDH1 *transcripts follow the general reduction in ribosome loading after nitrogen starvation and are unaltered in structure. This is in contrast to a group of transcripts with enhanced ribosome loading, namely *AMD2*, *ASP3*, *DAL5*, and *DAL7*, which all exhibit clear alterations in the 5' termini of their transcripts. The 5' end of the short form of *ASP3 *lies within the ORF, as was noted above for some of the pheromone-regulated transcripts. Two other transcripts, *UGA1 *and *MON1*, were found to have unaltered 5' termini after starvation, although they exhibit enhanced ribosome loading with nitrogen starvation.

### Influence of osmotic stress on the under-translated transcriptome

**Figure 7:**
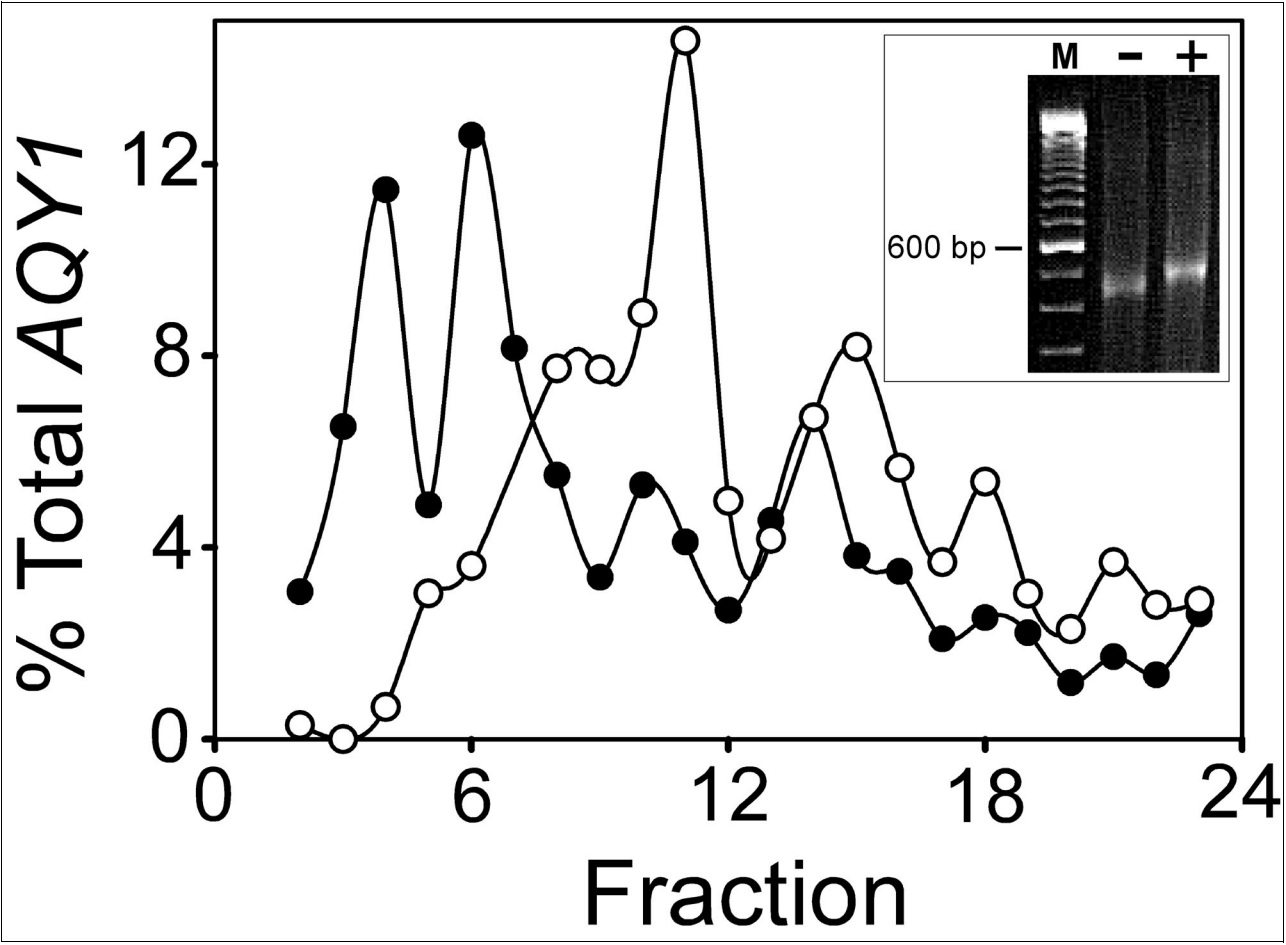
Response of *AQY1 *to osmotic stress. Cells were either grown in YPD (filled circles) or shocked by sorbitol addition for 30 minutes (open circles). Sucrose gradient centrifugation was performed and analyzed as described in Figure 2. The top of the gradient is to the left and the position of the 80S monosome is marked with the arrow. The 5' termini of the *AQY1 *transcript before and after osmotic stress are shown in the inset. Rapid amplification of cDNA ends (RACE) was carried out as described in the text (see Materials and methods), using as templates total RNA isolated from cells either grown in YPD (-) or osmotically shocked (+). The image contains the polymerase chain reaction products from the second RACE amplification step after separation by electrophoresis in 2% agarose gel. Lane 'M' contains a 100-basepair ladder.

Of the under-translated transcripts identified in growing cells, 18 were found to be related to responses to osmotic stress (Table 3). Total protein synthesis in osmotically stressed cells is inhibited [27,28], and this is reflected in a net decrease in polysome levels (not shown). Four of the genes included in Table 3, namely *AQY1*, *GCY1*, *HAL1*, and *PGM2*, exhibited an increase in ribosome loading in response to 1 mole per litre sorbitol. Figure 7 shows this increase in loading for *AQY1*. Analysis *of AQY1*, *GCY1*, and *PGM2 *by 5' RACE revealed a change in the 5' leader of *AQY1*, from within the ORF (+28) to -32 nucleotides relative to the initiator AUG (Figure 7, inset). In contrast there was no change in the structures of *GCY1 *and *PGM2 *(Table 1). Other workers found that the 5' terminus of *HAL1 *changes from -126 to a cluster from -38 to -68 (relative to the initiator AUG codon; Serrano R, Marques JA, personal communication). Thus, it appears that changes in ribosome loading in response to osmotic stress also can be accompanied by alterations in the transcript structure, as was observed with exposure to pheromone and nitrogen starvation.

## Discussion

Poorly translated cytosolic transcripts are usually found predominantly within mRNP particles [27] or with single ribosomes arrested on them [11], depending on the mechanism of regulation. Conversely, transcripts in the process of being translated into protein are generally associated with multiple actively translating ribosomes (polysomes). Because the average rate of movement of translating ribosomes along mRNAs (for example, polypeptide elongation) tends to be constant among different transcript species [28], it follows that the spacing of ribosomes along an mRNA is generally proportional to the rate of synthesis of the encoded protein. These considerations enable estimates of relative rates of synthesis of individual proteins across transcriptomes [1,3], which in turn allowed us to define a class of transcripts that are under-loaded with ribosomes and thus apparently translated at lower efficiencies than the majority of the transcriptome. However, this definition is not all-inclusive, because those transcripts whose translation is regulated through arrest of elongation would be located in the polysomal fraction and therefore would not be identified as under-translated by this analysis. Transcripts regulated at the level of polypeptide elongation may be prominent during early embryonic development [29,30] and among transcripts regulated by micro-RNAs [31]. Because of these considerations, the definition of less than 10% of the transcripts as 'under-translated' in growing yeast being may be an under-estimate.

### Mechanisms for generating alternate 5' untranslated regions

For a significant number of the genes implied to be under-translated during normal growth conditions, ribosome loading increased under the appropriate stress conditions, suggesting the existence of specific regulatory mechanisms that are responsive to environmental signals. One possible mechanism for this enhanced translation is suggested by the surprising frequency of regulated alterations in transcript structure. Of the 17 poorly loaded, translationally controlled transcripts examined in detail here, 12 exhibited structural changes coincident with altered ribosome loading in response to exogenous cues. The remaining five (*PRM4*, *UGA1*, *MON1*, *GCY1*, and *PGM2*) are likely to be solely under translational control.

The observed structural alterations were detected exclusively at the 5' ends of the transcripts. The sequences of 5' RACE products, together with RNase protection assays, demonstrated co-linearity between transcript and genomic sequences, providing no evidence for a regulated splicing mechanism similar to that involved in regulation of *HAC1 *in response to endoplasmic reticulum stress [32]. Excluding regulated splicing as a mechanism, the alternative forms seemingly arose either transcriptionally, through use of different promoters, or post-transcriptionally, either as normal intermediates of mRNA decay or through a new RNA cleavage mechanism. The requirement for *STE12 *revealed by this work points to a role for transcription in the pheromone-induced transcript changes described here, but this role could be direct or indirect. Consistent with a direct role for Ste12p-mediated promoter activation, TATA boxes and Ste12p binding sequences are found appropriately placed relative to the putative transcription starts of the pheromone-induced forms of the *HO*, *PRM2*, *PRY3*, and *SAG1 *transcripts (K.S. Bickel, unpublished observation). Previously, altered promoter usage was demonstrated directly for the nitrogen-regulated gene *CAN1 *and was suggested for *DAL5*, although the translatability of the alternative transcript forms was not assessed [33]. Promoter elements implicated in regulation of *CAN1 *and *DAL5 *are also found in the promoter regions of *AMD2 *and *DAL7*, suggesting the possibility of a similar switch in promoter usage.

Considering possible post-transcriptional mechanisms, the normal process of mRNA decay in the cytosol involves removal of the 5' cap, followed by 5'-3' exonucleolytic degradation [34]. A block to exonuclease action could produce some of the 5' truncated products described here. Perhaps related to this is that accumulation of 5' truncated transcripts in *Arabidopsis *was recently found to result from ribosome arrest mediated by nascent peptide [35]. Importantly, all known nonsplicing post-transcriptional mechanisms would be predicted to generate uncapped 5' ends, in contrast to the termini generated by RNA polymerase II initiation.

### Implications of altered 5'-untranslated regions for protein production

Because this is the first large-scale study relating ribosome loading to transcript structure, the frequency with which these regulated changes in transcript structure occur across nature is unknown. However, the suggestion that 9-18% of mammalian transcripts may have alternative first exons [12] is provocative. Two mammalian genes, in which alternative first exons were found to modify translation, are the gene encoding TIMP (tissue inhibitor of metalloproteinases) and the oncogene *mdm2*. With both of these genes, the translational efficiencies of the transcripts are regulated by changes in promoter utilization, which lead to altered 5' leaders [36,37].

In yeast, use of alternative promoters has been shown in some cases to produce different proteins. The *SUC2 *and *KAR4 *genes both contain multiple promoters, which generate different protein products with different biologic activities [38,39]. Similarly, the short forms of the *CRH1*, *KAR5*, *PRM2*, *PRP39*, *PRY3*, *ASP3*, and *AQY1 *mRNAs identified in this study lack the primary initiation codon, resulting in 5' truncated ORFs. These seven genes have the potential to create short protein products from internal AUG codons within the truncated mRNAs in the same ORFs as the primary products, although existence of these protein products has not been proven. With *PRM2*, *CRH1*, and *PRY3*, the putative amino-terminal truncated proteins lack signal sequences that target these three proteins to the endoplasmic reticulum. Therefore, if produced, the short protein products of these three genes probably differ in intracellular location, and possibly in function, from the full-length proteins. Similarly, the single transmembrane domain of the full-length Kar5 protein, which localizes it in the endoplasmic reticulum membrane, would be missing from the shorter, poorly translated form. These changes in protein targeting potentially could play roles in regulating cellular responses to pheromone.

With several other transcripts identified here - *HO*, *SAG1*, *AMD2*, *DAL5*, and *DAL7 *- the altered 5' leaders did not modify the protein encoding regions but profoundly altered the loading of ribosomes on the resulting transcripts. Although one can posit functional explanations for the truncated protein products produced from alternate transcripts, the biologic significance of 5' leaders with repressed translational activity is less obvious. The *HO *gene represents an extreme example in which a 5' leader as long as 2 kilobases is produced in response to pheromone treatment and the long transcripts are located primarily in untranslated mRNP particles. Likewise, poor translation of the *SAG1 *transcript in growing cells is mediated by an inhibitory 826-nucleotide 5' UTR. A similar situation seems to occur with the nitrogen-regulated *AMD2*, *DAL5*, and *DAL7 *transcripts. In addition to the changes in ribosome loading observed here, the levels of all five of these transcripts are regulated at the transcriptional level. One outcome of these parallel changes in transcript level and translation efficiency is to amplify the biologic consequence of transcriptional control by accentuating the upregulation or downregulation of protein production. This is surely one mechanism for the 'homodirectional' changes in transcript level and ribosome loading that have been noted by others on a global level in yeast [2]. However, this rationalization neglects the conundrum of why the cell does not simply enhance an expression response by switching transcription completely off.

### Implications for transcriptional mechanisms

Why should the cell produce a transcript that is either poorly translated or not translated at all? One speculative role for the continued synthesis of translationally inactive transcripts, under conditions in which the protein product is not needed, could involve regulation of accessibility to the promoter regions of these genes. One suggested role for the 'intergenic' transcription, which has been found widely in eukaryotes [40,41], is to assist in maintaining an open chromatin state required for facile transcriptional activation. Intergenic transcription has been found in the locus control regions of the mammalian ß-globin and MHC (major histocompatibility complex) class II loci [42,43], in the promoter regions of the interleukin-4 and interleukin-13 genes [44], in the V(D)J region of the mouse immunoglobulin heavy chain locus [45] and within the *Drosophila *bithorax complex [46]. RNA polymerase II is found upstream of many apparently inactive genes in stationary phase *S. cerevisiae *[47]. It is noteworthy that the 5' leader of the long *HO *transcript extends 2,000 nucleotides upstream of the coding region through a region that is devoid of genes (for example, intergenic) and which contains a multitude of transcription factor binding sites that mediate the complex transcriptional control of the *HO *gene (discussed by Krebs and coworkers [48]). Maintenance of this extended region in an open state through continued low level transcription of the translationally inactive transcript species could allow rapid reactivation of *HO *transcription upon removal of pheromone.

In addition to keeping chromatin in an active state, transcription from intergenic regions can also be involved in repressing transcription from promoters. This has been found to occur either by local competition between promoters [49] or through interference by elongating polymerases coming from an upstream promoter [50]. The competition model applies equally to promoters located upstream or downstream of the primary promoter, which is consistent with the occurrence of both longer and shorter 5' UTRs in this study.

Very little is known of the mechanisms that prevent inappropriate protein production from intergenic transcripts. Some 'cryptic' RNA polymerase II products are removed within the nucleus through a highly conserved process utilizing a unique poly(A) polymerase and the nuclear exosome [51]. Nonsense-mediated decay [52,53], another highly conserved process [54,55], removes those transcripts that are recognized as having premature translation termination codons. This paper describes a third process, translational silencing, wherein continuing synthesis of transcripts with inhibitory 5' leaders contributes to an open chromatin structure while protecting the cell from inappropriate protein production. At this time, we have no evidence defining the inhibitory elements in the 5' leaders of the silenced transcripts. As discussed in the Background section (above), possible inhibitory features could be secondary structure, protein binding sites, or ATG codons upstream of the coding region. With regard to the latter mechanism, we have noted ATG sequences in all of the long, inhibitory 5' leaders. For example, the long forms of the *DAL5 *and *AMD2 *5' leaders contain five and two ATG codons, respectively, whereas neither short form contains an ATG upstream of the start codon. Further experimental work will be required to establish the inhibitory elements in the translationally silenced transcripts.

## Materials and methods

### Yeast cultures and polysome fractionation

All experiments used strain BY2125 (MATa *ade2-1 his3-11,15 leu2-3,112 ura3-1 can1-100 ssd1-d *: W303 background). Strains VM1906 (Δfus3::LEU2 Δkss1::TRP1), VM1718 (Δste12::TRP1) and LL1 (Δgcn2::TRP1) were derived from BY2125 by gene disruption [56].

Cells were grown at 30°C in rich glucose medium, YPD (1% yeast extract, 2% peptone and 2% glucose) [57], to mid-log phase (approximately 1 × 10^7 ^cells/ml) before harvesting. Preparations of cell lysates and polysome fractionation were described previously, as was pheromone treatment of yeast cultures [3]. For nitrogen starvation, cultures were grown at 30°C in minimal glucose medium [57] with necessary supplements to mid-log phase, washed once with 10 mmol/l potassium phosphate (pH 7.0), and then incubated for 30 minutes at 30°C in pre-warmed nitrogen starvation medium (0.2% yeast nitrogen base [without amino acids or ammonium sulfate], 3% glucose, 20 mmol/l potassium phosphate [pH 7.0], and adenine and uracil added at 40 and 20 µg/ml, respectively) [58]. For osmotic stress, exponential phase YPD cultures (approximately 8 × 10^6 ^cells/ml) were diluted into an equal volume of pre-warmed YPD or YPD + 2 mol/l sorbitol. Incubation at 30°C was continued for 30 minutes before the cultures were harvested.

### RNA analysis

RNA was isolated using Qiagen RNeasy mini-columns (Qiagen Corp., Valencia, CA, USA). An equal proportion of the RNA isolated from each sucrose gradient fraction was used directly in reverse transcription reactions using anchored oligo(dT)_25 _primers. When comparing changes in total RNA isolated from different culture conditions or treatments, equal quantities of total RNA were used for reverse transcription reactions. QPCR was performed as described previously [3].

Northern blot analysis followed a procedure described previously [59], as did the RNase protection assays [60]. The *SAG1 *RNase protection assay probe was 631 bases long and contained 484 nucleotides 5' of the coding region and 55 nucleotides into the coding region. 5' RACE was carried out as described by Frohman [61] using gene specific primers for the reverse transcription reaction. The Thermoscript RT-PCR system (Invitrogen, Carlsbad, CA, USA) was used allowing for the reverse transcription reaction to be done at 55°C to minimize reverse transcriptase stops due to secondary structure in the RNA. To estimate the *HO *5' leader in cells treated with a-factor for 30 minutes, reverse transcription reactions were done as described above and the products were used as DNA template in a series of PCR reactions. Two reverse primers, located -500 and -1493 nucleotides relative to the initiation codon of the *HO *ORF and 10 different forward primers, spaced roughly 200 nucleotides apart starting at -700, were used.

### Determination of ribosome loading ratio

Using QPCR, relative levels of mRNA across polysome gradients were determined for the indicated genes in growing cells or treated cells. The treatment was either pheromone treatment for 30 minutes, nitrogen starvation for 30 minutes or osmotic stress for 30 minutes. Using the Abs_260 nm _traces from the polysome gradients, the number of ribosomes associated with a specific mRNA in each fraction was determined. The ribosome loading ratio was calculated by dividing the average number of ribosomes associated with a transcript from a polysome gradient from treated cells divided by the average number of ribosomes associated with a transcript from a polysome gradient from growing cells. A number greater than 1 indicates an increase in ribosome loading with treatment and conversely a number less than 1 indicates a decrease in ribosome loading with treatment.

### Construction of *HIS3-HA *reporter plasmids

A *HIS3-HA *reporter plasmid (pVW12) was constructed by insertion of the *HIS3-HA *sequence from pVW06 [3] between the *Bam *HI and *Eco *RI sites of the multiple cloning sequence of plasmid pRS416ADH1p [62], so that *HIS3-HA *transcription is from the constitutive *ADH1 *promoter. The *ADH1 *5' leader in pVW12 (nucleotides -48 to -1) was replaced with either the *SAG1 *short 5' leader (nucleotides -48 to -1, plasmid pVW13) or the *SAG1 *long 5' leader (nucleotides -836 to -1, plasmid pVW14) using plasmid gap repair [63]. Specifically, pVW12 was cleaved in the 5' leader with *Spe *I and *Xba *I and transformed into strain BY2125 with PCR fragments bearing the short or long *SAG1 *5' leader flanked with 5' and 3' sequences homologous to the *ADH1 *promoter (-91 to -49) and *HIS3-HA *(+1 to +48). Ura^+ ^yeast transformants were screened by PCR to identify plasmids repaired with the *SAG1 *fragments and confirmed by DNA sequencing. S1 nuclease protection assays were carried out as described [64,65], using gel purified oligonucleotides (Qiagen Corp.), on RNA isolated from pVW13 or pVW14 to confirm the 5' ends of each transcript.

### Western blots

Yeast transformed with pRS416ADH1, pVW13, or pVW14 were grown in selective medium (synthetic complete medium with casamino acids and lacking uracil) to mid-exponential phase, harvested, and lysed as described previously [3]. Protein samples (5 µg for the pVW13 lysate and 50 µg each for the pVW14 and pRS416ADH1p lysates) were separated by electrophoresis on a 10% polyacrylamide gel and transferred electrophoretically to PVDF membrane. The membrane was incubated with anti-HA mouse monoclonal antibody HA.11 (Covance Research Products, Berkeley, CA, USA) and sheep anti-mouse immunoglobulin conjugated with horseradish peroxidase (Amersham Biosciences, Piscataway, NJ, USA), then developed with ECL Plus Western Blotting Detection System (Amersham Biosciences). His-HA protein was quantitated using a Storm 840 phosphorimager (Amersham Biosciences).

## Additional data files

The following additional data are included with the online version of this article: A text file containing the data used to construct Figure 1, parts a and b (Additional data file 1); a text file containing the data used to construct Figure 1, parts c (Additional data file 2); and a text file containing the data used to determine ribosome loading ratio in Figure 1 (Additional data file 3).

## Supplementary Material

Additional data file 1 A text file containing the data used to construct Figure 1 parts a and b Click here for file

Additional data file 2 A text file containing the data used to construct Figure 1 part c Click here for file

Additional data file 3 A text file containing the data used to determine ribosome loading ratio in Figure 1 Click here for file

## Figures and Tables

**Table 1:** Influence of pheromone treatment, nitrogen starvation and osmotic stress on 5' leader structure and ribosome loading

	5' termini		
		
Gene	Steady state	Treated	Loading ratio
Pheromone response			
***CRH1 ***	-80	+2,+54	0.6
***HO ***	-39	approx. -2,000	0.3
***KAR5 ***	+115,+166	-2	5.9
***PRM2 ***	+94,+297	-45	6.1
***PRP39 ***	-89	approx. +300	0.3
***PRY3 ***	-76	+452	0.6
***SAG1 ***	-826	-38	4.2
*PRM4 *	-64	-64	1.9
*BAR1 *^b^	-52	-52	1.0^a^
*FAR1 *^b^	-47	-47	1.0^a^
*STE2 *^b^	-31	-31	1.0^a^
Nitrogen starvation			
***AMD2 ***	-97	-23	2.7
***ASP3 ***	+657	-22	24.4
***DAL5 ***	-273	-53	15.0
***DAL7 ***	-159	-26	7.0
*UGA1 *	-38	-38	2.0
*MON1 *	-35	-35	1.8
*ASP1 *^b^	-41	-41	0.3
*GDH1 *^b^	-67	-67	0.7
Osmotic stress			
***AQY1 ***	+28	-32	1.9
*GCY1 *	-58	-58	2.6
*PGM2 *	-60	-60	1.9

The 5' termini of the transcripts are expressed as nucleotides relative to the initiation codon of the open reading frame (ORF) and were determined by 5' rapid amplification of cDNA ends (RACE), except for *HO *and *PRP39 *in pheromone-treated cells, which were estimated from Northern blots and polymerase chain reaction walking. Ribosome loading is defined as the average number of ribosomes associated with a transcript and was determined as outlined in the method section except when indicated differently. The genes that exhibit a change in 5' untranslated region upon treatment are presented in bold font. The nitrogen starvation experiments were carried out with the ?*gcn2 *strain. ^a^These values were calculated from the data presented by MacKay and coworkers [3]. ^b^These are control genes that do not change in ribosome loading.

**Table 2:** Under-translated genes involved in responses to nitrogen stress

Gene	Function
*AMD2 *	Amidase
*APG13 *	Autophagy
*APG5 *	Autophagy
*ARG80 *	Regulation of arginine and ornithine utilization
*ARO80 *	Regulation of aromatic amino acid catabolism
*ASP3 *	Asparaginase
*CCZ1 *	Autophagy
*DAL5 *	Allantoate metabolism
*DAL7 *	Allantoate metabolism
*DOA4 *	Regulates amino acid permease Gap1p
*GCN4 *	General control of amino acid biosynthesis
*GDH2 *	Glutamate dehydrogenase
*GZF3 *	Regulates nitrogen catabolic gene expression
*LST4 *	Regulates amino acid permease Gap1p
*MON1 *	Autophagy
*MUP3 *	Methionine permease
*STP2 *	Regulator of amino acid permease genes
*VPS30 *	Autophagy
*UGA1 *	GABA aminotransferase
*YSP3 *	Peptidase

This list of genes was derived from a Gene Ontology analysis of the translation state of transcripts of yeast cells growing in rich-glucose medium [3].

**Table 3:** Under-translated osmoregulatory genes

Gene	Function
*GCY1 *	Salt induced aldo-keto reductase
*AQY1 *	Aquaporin
*ALD3 *	Aldehyde dehydrogenase, activity increased by osmotic shock
*BCK1 *	MAPKKK in the PKC pathway
*HAL1 *	Halotolerance
*MSN1 *	Present with Hot1p at GPD1 promoter only during osmostress
*HAL5 *	Cation homeostasis
*HOT1 *	Transcription factor, high osmolarity
*NST1 *	Negative effector of halotolerance
*SSK22 *	MAPKK osmosensing, redundant w/SSK2
*SSK1 *	osmosensing activator of MAPK pathway
*SSK2 *	MAPKK osmosensing
*DOA4 *	Involved in vacuole biogenesis and osmoregulation
*HOG1 *	MAPK in osmolarity response
*DAK2 *	Glycerone kinase, response to stress
*APA2 *	Osmoregulation in vacuole
*PGM2 *	Osmoregulation
*WSC3 *	Osmoregulation

This list of genes was derived from a Gene Ontology analysis of the translation state of transcripts of yeast cells growing in rich-glucose medium [3]. MAPK, mitogen-activated protein kinase; MAPKK, MAPK kinase; MAPKKK, MAPK kinase kinase; PKC, protein kinase C.
